# Evaluation of AQP4 functional variants and its association with fragile X-associated tremor/ataxia syndrome

**DOI:** 10.3389/fnagi.2022.1073258

**Published:** 2023-01-06

**Authors:** Andrea Elias-Mas, Miriam Potrony, Jaume Bague, David J. Cutler, Maria Isabel Alvarez-Mora, Teresa Torres, Tamara Barcos, Joan Anton Puig-Butille, Marta Rubio, Irene Madrigal, Susana Puig, Emily G. Allen, Laia Rodriguez-Revenga

**Affiliations:** ^1^Radiology Department, Hospital Universitari Mútua de Terrassa, Terrassa, Barcelona, Spain; ^2^Institute for Research and Innovation Parc Taulí (I3PT), Sabadell, Spain; ^3^Genetics Doctorate Program, Universitat de Barcelona (UB), Barcelona, Spain; ^4^Biochemistry and Molecular Genetics Department, Hospital Clinic of Barcelona, Barcelona, Spain; ^5^Institut d’Investigacions Biomèdiques August Pi i Sunyer (IDIBAPS), Barcelona, Spain; ^6^CIBER of Rare Diseases (CIBERER), Instituto de Salud Carlos III, Barcelona, Spain; ^7^Dermatology Department, Melanoma Unit, Hospital Clínic de Barcelona, Universitat de Barcelona, Barcelona, Spain; ^8^Department of Human Genetics, Emory University School of Medicine, Atlanta, GA, United States; ^9^Molecular Biology CORE, Hospital Clinic of Barcelona, Barcelona, Spain; ^10^Department of Neurology, Parc Taulí Hospital Universitari, Sabadell, Spain

**Keywords:** FXTAS, *AQP4*, *FMR1* premutation, genetic variation, glymphatic system

## Abstract

**Introduction:**

Fragile X-associated tremor/ataxia syndrome (FXTAS, OMIM# 300623) is a late-onset neurodegenerative disorder with reduced penetrance that appears in adult *FMR1* premutation carriers (55–200 CGGs). Clinical symptoms in FXTAS patients usually begin with an action tremor. After that, different findings including ataxia, and more variably, loss of sensation in the distal lower extremities and autonomic dysfunction, may occur, and gradually progress. Cognitive deficits are also observed, and include memory problems and executive function deficits, with a gradual progression to dementia in some individuals. Aquaporin 4 (AQP4) is a commonly distributed water channel in astrocytes of the central nervous system. Changes in AQP4 activity and expression have been implicated in several central nervous system disorders. Previous studies have suggested the associations of *AQP4* single nucleotide polymorphisms (SNPs) with brain-water homeostasis, and neurodegeneration disease. To date, this association has not been studied in FXTAS.

**Methods:**

To investigate the association of *AQP4* SNPs with the risk of presenting FXTAS, a total of seven common *AQP4* SNPs were selected and genotyped in 95 *FMR1* premutation carriers with FXTAS and in 65 *FMR1* premutation carriers without FXTAS.

**Results:**

The frequency of *AQP4*-haplotype was compared between groups, denoting 26 heterozygous individuals and 5 homozygotes as carriers of the minor allele in the FXTAS group and 25 heterozygous and 2 homozygotes in the no-FXTAS group. Statistical analyses showed no significant associations between *AQP4* SNPs/haplotypes and development of FXTAS.

**Discussion:**

Although *AQP4* has been implicated in a wide range of brain disorders, its involvement in FXTAS remains unclear. The identification of novel genetic markers predisposing to FXTAS or modulating disease progression is critical for future research involving predictors and treatments.

## Introduction

The brain is a high-energy consuming organ with a high metabolic activity, producing a substantial amount of interstitial waste products. Efficient clearance of the brain’s metabolic waste is needed in order to avoid their accumulation, causing several neurological diseases (Kaur et al., 2021). Since there is a lack of conventional lymphoid circulation in the brain, the glymphatic system has been postulated as an alternative clearance for the brain waste product (Iliff et al., 2015), though evidence is still incomplete (Hladky and Barrand, 2022).

In the glymphatic system, cerebrospinal fluid (CSF) flows into the brain parenchyma within the periarterial spaces that surround the penetrating cerebral arteries, also called the perivascular spaces. Facilitated by aquaporin 4 (AQP4), CSF flows from the periarterial space into the brain interstitium and mixes with interstitial fluid, which, along with interstitial solutes, travels into the perivenous spaces, draining the fluid and its contents into the deep veins and into the basal meningeal and cervical lymphatic vessels (Hablitz and Nedergaard, 2021). AQP4 is the most abundant water channel in the brain, and, since it has a role regulating fluid exchange between perivascular spaces and the rest of the glymphatic system, it is considered the most important element in it (Nagelhus and Ottersen, 2013; Papadopoulos and Verkman, 2013; Szczygielski et al., 2021).

Alterations of glymphatic fluid circulation through AQPs variations are now emerging as central elements in the pathophysiology of different brain conditions. In fact, dysfunction of AQP4 have been implicated in the pathogenesis of many degenerative disorders, including Alzheimer’s disease (AD), vascular cognitive impairment, idiopathic normal-pressure hydrocephalus, Parkinson’s disease dementia, frontotemporal dementia and Creutzfeldt-Jakob disease (Zeppenfeld et al., 2017; Nedergaard and Goldman, 2020; Silva et al., 2021; Wang et al., 2022). Furthermore, evidence indicates that genetic variation in *AQP4* modulates sleep quality and architecture, amyloid-β burden and rate and progression of cognitive decline in AD patients (Burfeind et al., 2017; Rainey-Smith et al., 2018; Ulv Larsen et al., 2020). Despite the relationship between glymphatic dysfunction and neurodegenerative diseases, dysfunction of glymphatic system has not yet been studied in Fragile X-associated tremor/ataxia syndrome (FXTAS) and its association with *AQP4* genetic variants is unknown. FXTAS is a neurodegenerative disorder linked to *FMR1* gene premutation carriers (55–200 CGG repeats) that is associated with cognitive loss that can evolve into dementia. Intranuclear inclusions and increased β amyloid load has been discovered in brains of patients with FXTAS (Cabal-Herrera et al., 2020; Salcedo-Arellano et al., 2021a). On the basis of these observations, we analyzed *AQP4* functional variants with the aim to investigate whether AQP4 could be a new genetic risk factor for FXTAS.

## Materials and methods

### Study population

The present study was conducted on genotype data from a total of 160 unrelated *FMR1* premutation carriers (95 presenting FXTAS symptoms and 65 without FXTAS clinical symptoms). Participants were identified through previous fragile X syndrome research projects at Emory University (Atlanta, GA, USA), through recruitment efforts at scientific conferences, and through collaborations with other research groups. All participants were enrolled from families with members known to be affected with fragile X-associated conditions and molecularly diagnosed. Table 1 summarizes general demographics of the participants. FXTAS subjects were screened for eligibility as described in Kong et al. (2022). Briefly, case subjects were male or female premutation carriers with symptoms of tremor or ataxia before age 65, as reviewed by a neurologist. Control individuals, named as the no-FXTAS group, were male premutation carriers that reached age 68 without significant tremor or ataxia symptoms, as reviewed by a neurologist. The protocols and consent forms were approved by the Institutional Review Board at Emory University, and written informed consent was obtained from all subjects (IRB00074941).

**TABLE 1:** Description of the individuals recruited in this study.

	FXTAS cases (*n* = 95)	No-FXTAS cases (*n* = 65)
Males/female [no (%)]	80 (84%)/15 (16%)	65 (100%)/0
Age (mean ± SD, *Y*)*	67.70 ± 10.81	77.01 ± 6.38
Age min, max (*Y*)	27–94	66–98
CGG repeat size (mean ± SD)*	93.15 ± 20.33	75.00 ± 15.14
CGG repeat size min, max	55–150	56–150

*p < 0.0001 using Student’s *t*-test for comparison of means.

### SNPs of the *AQP4* gene and haplotype analysis

Seven tag single nucleotide polymorphisms (SNPs) across *AQP4* gene (NM_001650) were selected according to their location and known functions, based on earlier reports on their associations with clinical phenotypes (Ulv Larsen et al., 2020). The SNPs were considered for those above 15% in Utah Residents with Northern and Western European Ancestry (CEU) population according to minor allele frequency (MAF: 0.15∼0.26). These SNPs included rs162007 (Chr18:26865883, Upstream, MAF 0.16), rs162008 (Chr18:26865728, 5′UTR, MAF 0.20), rs63514 (Chr18:26863457, intron, MAF 0.17), rs335931 (Chr18:26859108, intron, MAF 0.15), rs335930 (Chr18:26856961, intron, MAF 0.23), rs335929 (Chr18:26855623, 3′UTR, MAF 0.14), rs16942851 (Chr18:26851725, downstream, MAF 0.14). The chromosome positions are based on hg38.

### Genotyping of *AQP4* SNPs

Whole genome sequencing was performed on samples using Illumina platforms at Hudson Alpha or Novogene as described in Kong et al. (2022). All samples were mapped using PEMapper and called using PECaller (Johnston et al., 2017). Genomic data have been uploaded to the National Institute of Mental Health (NIMH) Data Archive.^1^

### Statistical analysis

To test the population homogeneity of the study subjects, the allele frequencies were tested against Hardy-Weinberg equilibrium (HWE) by the χ2-test. The plink v1.07 toolset was used to perform SNP association and haplotype^2^ (Purcell et al., 2007). The power analysis was performed using the ‘‘Quanto’’ tool.^3^ Statistical analyses were performed using commercially available software (SPSS SmartViewer, version 18.0; SPSS, Chicago, IL, USA). *P*-values < 0.05 were considered statistically significant. Association tests were corrected using the Benjamini and Hochberg step-up False Discovery Rate (FDR) for multiple comparisons.

## Results

Genotype data from 95 FXTAS and 65 no-FXTAS individuals were analyzed. Table 1 shows the demographic data of FXTAS and no-FXTAS group. Significant differences were found for the age and the CGG repeat size when comparing both groups (*p* < 0.0001 and *p* < 0.0001, respectively). Age difference can be attributed to a bias in the recruitment of no-FXTAS individuals. In order to make sure that *FMR1* permutations in the no-FXTAS group did not have neurologic symptoms older men were included in this cohort. As for the CGG repeat size, the difference found might account for the CGG-repeat dependence described in clinical and neuropathologic features of FXTAS.

In agreement with HWE, there was no deviation detected in any of the analyzed SNPs (*p* > 0.3). All SNPs studied were in linkage disequilibrium (LD), and the pairwise LD coefficient (*r*^2^) ranged between 0.8 and 1. Table 2 shows the genotype frequency for each SNP according to the presence of FXTAS symptoms. After correction of *p*-values for multiple comparisons, there was no significant difference in frequencies of any of the analyzed SNPs between FXTAS and no-FXTAS group (Table 3). Adjustment for sex did not change these results (data not shown). Table 4 shows the results of the genotype association analysis between *AQP4* polymorphisms and risk of FXTAS, according to different genetic inheritance models.

**TABLE 2:** Genotype frequency for each SNP according to presence of FXTAS.

	Genotype	FXTAS (*n* = 95)	No-FXTAS (*n* = 65)
rs162007	GG	64 (67.4%)	37 (56.9%)
	GA	26 (27.3%)	25 (38.5%)
	AA	5 (5.3%)	3 (4.6%)
rs162008	CC	64 (67.4%)	36 (55.4%)
	CT	26 (27.3%)	26 (40%)
	TT	5 (5.3%)	3 (4.6%)
rs63514	CC	63 (66.3%)	37 (56.9%)
	CT	27 (28.4%)	25 (38.5%)
	TT	5 (5.3%)	3 (4.6%)
rs335931	AA	63 (66.3%)	38 (58.5%)
	AG	27 (28.4%)	25 (38.5%)
	GG	5 (5.3%)	2 (3%)
rs335930	AA	57 (60%)	36 (55.4%)
	AC	32 (33.7%)	26 (40%)
	CC	6 (6.3)	3 (4.6%)
rs335929	AA	63 (66.3%)	38 (58.5%)
	AC	27 (28.4%)	24 (36.9%)
	CC	5 (5.3%)	3 (4.6%)
rs16942851	TT	64 (67.4%)	38 (58.5%)
	TG	26 (27.3%)	24 (36.9%)
	GG	5 (5.3%)	3 (4.6%)

**TABLE 3:** Single nucleotide polymorphism (SNP) allele association analysis.

SNP	FXTAS MAF	No-FXTAS MAF	OR (95% CI)	*P*-value	Adj *P*-value
rs16942851	0.19	0.22	0.81 (0.47–1.41)	0.463	0.628
rs335929	0.19	0.22	0.84 (0.49–1.46)	0.538	0.628
rs335930	0.23	0.24	0.96 (0.57–1.63)	0.887	0.887
rs335931	0.19	0.22	0.84 (0.49–1.46)	0.538	0.628
rs63514	0.19	0.24	0.77 (0.45–1.33)	0.348	0.628
rs162008	0.19	0.25	0.72 (0.42–1.23)	0.224	0.628
rs162007	0.19	0.24	0.75 (0.43–1.28)	0.290	0.628

MAF, minor allele frequency; OR, odd-ratio. Adj *p*-value, adjusted *p*-value using Benjamini and Hochberg step-up false discovery rate, for multiple comparisons.

**TABLE 4:** Genotype association using different genetic models.

SNP	Allele	Test	OR (95% CI)	*P*-value	Adj *P*-value
rs16942851	G	ADD	1.22 (0.52–2.83)	0.647	0.978
		DOM	0.68 (0.35–1.31)	0.251	0.365
		REC	1.75 (0.33–9.31)	0.512	0.854
rs335929	C	ADD	1.23 (0.53–2.86)	0.634	0.978
		DOM	0.71 (0.37–1.37)	0.313	0.365
		REC	1.75 (0.33–9.31)	0.512	0.854
rs335930	C	ADD	1.38 (0.60–3.15)	0.449	0.978
		DOM	0.83 (0.43–1.57)	0.561	0.561
		REC	2.12 (0.41–10.87)	0.366	0.854
rs335931	G	ADD	1.23 (0.53–2.86)	0.634	0.978
		DOM	0.71 (0.37–1.37)	0.313	0.365
		REC	1.75 (0.33–9.31)	0.512	0.854
rs63514	T	ADD	0.99 (0.47–2.08)	0.978	0.978
		DOM	0.67 (0.35–1.29)	0.229	0.365
		REC	1.15 (0.26–4.98)	0.854	0.854
rs162008	T	ADD	0.97 (0.46–2.04)	0.932	0.978
		DOM	0.60 (0.31–1.15)	0.125	0.365
		REC	1.15 (0.26–4.98)	0.854	0.854
rs162007	A	ADD	0.98 (0.47–2.06)	0.961	0.978
		DOM	0.64 (0.33–1.23)	0.180	0.365
		REC	1.15 (0.26–4.98)	0.854	0.854

ADD, additive model; DOM, dominant model; REC, recessive model. Adj *p*-value, adjusted *p*-value using Benjamini and Hochberg step-up false discovery rate, for multiple comparisons.

## Discussion

Fragile X-associated tremor/ataxia syndrome is a late-onset neurodegenerative disorder with reduced penetrance, meaning that not all *FMR1* premutation carriers will develop it (Hagerman et al., 2001). Among *FMR1* premutation carriers older than 50 years, it has been estimated that 40% of men and 16% of women will develop FXTAS symptoms, although there is significant variability in the progression of neurological dysfunction (Coffey et al., 2008; Rodriguez-Revenga et al., 2009). The description and characterization of FXTAS is of great interest, because the prevalence of *FMR1* premutation in the general population is relatively high. It has been estimated that *FMR1* premutation affects ∼1 out of 400 males and 1 out of 200 females (Tassone et al., 2012), leading to symptoms of FXTAS in up to 1 in 3000 men older than 50 years. Even though the *FMR1* premutation is the major risk factor for FXTAS, there are still some unknown genetic, epigenetic or environmental factors that might be affecting gene penetrance. Candidate gene SNP association analysis is a commonly used approach to identify risk alleles and their association with clinical traits. In the present study we selected this method to investigate the role of *AQP4* gene variants in FXTAS susceptibility. We hypothesized that *AQP4* polymorphisms could play a role as risk factors for FXTAS. However, we did not find any significant difference in the distributions of alleles, genotypes, and haplotypes between FXTAS and no-FXTAS individuals, after correction for multiple testing.

A myriad of different studies point out *AQP4* gene as a novel candidate gene for brain plasticity and associated with neuropsychiatric and neurodegenerative disorders. According to the human postmortem brain microarray data from the Allen Brain Atlas resources,^4^ *AQP4* is most highly expressed in fronto-limbic and temporal cortical regions. Both neuroanatomical areas are linked to cognitive and executive processes, and its disturbance leads to the neuropsychological changes described in many different movement disorders (Robertson et al., 2016). Although indirectly, genome-wide linkage studies have repeatedly pointed out the role of *AQP4* in the development of brain disorders (Dadgostar et al., 2021). Genetic variations, abnormal distribution and quantitative abnormalities of *AQP4* have also been associated with several neurodegenerative disorders, such as AD, Parkinson’s disease and amyotrophic lateral sclerosis (reviewed in Mader and Brimberg, 2019). Recently the rs162008, the most prevalent genetic variant of *AQP4*, has been associated with a ∼15% change in AQP4 expression. In AD, genetic variations in *AQP4* were shown to be associated with changes in sleep pattern and increased β-amyloid (Rainey-Smith et al., 2018), as well as to β-amyloid accumulation and disease stage progression (Burfeind et al., 2017; Chandra et al., 2021). Taking everything into account, it is implied that AQP4 distribution and regulation might have crucial role in neuronal activity and function.

Apart from intention tremor and cerebellar ataxia, core clinical features of FXTAS include executive dysfunction which may progress to dementia in some cases (Hagerman et al., 2001; Hall et al., 2016). In addition, several conditions affecting sleep quality have been frequently described among FXATS patients (Hamlin et al., 2011; Summers et al., 2014). FXTAS can coexist with other neurodegenerative disorders, such as Parkinson’s disease and AD (Aydin et al., 2020; Salcedo-Arellano et al., 2021b), suggesting a synergistic effect on the progression of disease symptoms. On the basis of these observations and the evidence of the consequences of AQP4 dysfunction in neurological conditions, we analyzed genetic variation of *AQP4* gene among FXTAS patients. We compared frequency of alleles, genotypes, and haplotypes of AQP4 between FXTAs and no-FXTAS cases. Results did not find any association between any of the SNPs analyzed and the risk of developing FXTAS (Table 4). Furthermore, no association was detected when comparing frequency distribution of the two major *AQP4*-haplotypes. In fact, the frequency detected did not differ from the one described among CEU population (Table 5). Although no relationship between genetic variants in *AQP4* gene and FXTAS was found, no association with changes in the development of the disease has been assessed due to lack of clinical information. Similarly with the AD, *AQP4* SNPs have been associated with some aspects of the clinical course, such as faster cognitive decline, rather than the presence or absence of the disease (Burfeind et al., 2017).

**TABLE 5:** Major allele and minor allele haplotype (HTMa and HTMi) frequencies of *AQP4* functional SNPs across the CEU (Utah residents with ancestry from Northern and Western Europe) population and the FXTAS and no-FXTAS groups.

	Haplotype frequency CEU	FXTAS (*n* = 190)	No-FXTAS (*n* = 130)
HTMa	0.767	0.76842105	0.73846154
HTMi	0.1966	0.18947368	0.22307692

As previously described (Ulv Larsen et al., 2020), examination of the SNPs revealed two conserved haplotypes: HtMa (haplotype for the major allele) and HtMi (haplotype for the minor allele). Haplotype frequency comparison by means of dominant analysis between FXTAS and no-FXTAS group did not show significant differences (*p* > 0.05). Moreover, both groups showed similar haplotype frequency compared to the CEU population (Table 5).

Given the sample size this study had limited power. *Post hoc* power analyses showed that the power to detect the observed odds ratios for FXTAS cases vs. no-FXTAS ranged from 0.05 to 0.19.

This study has two main limitations that might be explained because of the rarity of the disorder. First the relatively small sample size. Power ranged only from 0.05 to 0.19. Second, the age differences between groups and the fact that those individuals considered as no-FXTAS may develop clinical symptoms later in life, masking differences among groups. Nonetheless, to our knowledge we are reporting for the first time, that the *AQP4* SNPs (rs162007, rs162008, rs63514, rs335931, rs335930, rs335929, and rs16942851) and haplotypes were not associated with susceptibility of FXTAS in Caucasian population. Despite this lack of association, further studies are necessary to fully discard the role of AQP4 and glymphatic system in the pathology of FXTAS. There is a need to describe new evidence into how the glymphatic system functions, and how AQP4 dysfunction might take part into FXTAS disease progression.

## Data availability statement

The original contributions presented in this study are included in the article, further inquiries can be directed to the corresponding authors.

## Ethics statement

The studies involving human participants were reviewed and approved by the Institutional Review Board at Emory University (IRB00074941). The patients/participants provided their written informed consent to participate in this study.

## Author contributions

All authors listed have made a substantial, direct, and intellectual contribution to the work, and approved it for publication.

## Footnotes

1
https://nda.nih.gov/edit_collection.html?id=2380

2
https://zzz.bwh.harvard.edu/plink

3
https://bio.tools/QUANTO

4
http://www.brain-map.org
